# A presentation of postcardiac injury syndrome after successful chronic total occlusion percutaneous coronary intervention using dissection re‐entry techniques

**DOI:** 10.1002/ccr3.955

**Published:** 2017-04-18

**Authors:** Gabby Elbaz‐Greener, Harindra C. Wijeysundera

**Affiliations:** ^1^ Schulich Heart CenterSunnybrook Health Sciences CenterUniversity of TorontoTorontoOntarioCanada

**Keywords:** Case report, chronic total occlusion, pericardial effusion, pleural effusion

## Abstract

Retrograde dissection re‐entry can cause pericardial trauma of sufficient degree to lead to the development of an auto‐immune postpericardial injury syndrome. Clinical suspicion for this condition should be high in the event of fever, symptoms, pericardial/pleural effusion, and pleuritic chest pain following chronic total occlusion (CTO) Post cardiac injury syndromes (PCI).

## Introduction

Postcardiac injury syndrome (PCIS) is an umbrella term for inflammatory pericardial syndromes including postmyocardial infarction pericarditis (Dressler's syndrome), postpericardiotomy syndrome (PPS), and post‐traumatic pericarditis 1, 2. PCIS after open‐heart surgery is common with an incidence of 10–50% 3. However, the incidence of pericardial complications after percutaneous coronary intervention (PCI) is less than 0.2% 4, 5. We present a case of acute pericarditis, which occurred after a successful complex PCI.

## Case report

**Figure 1:**
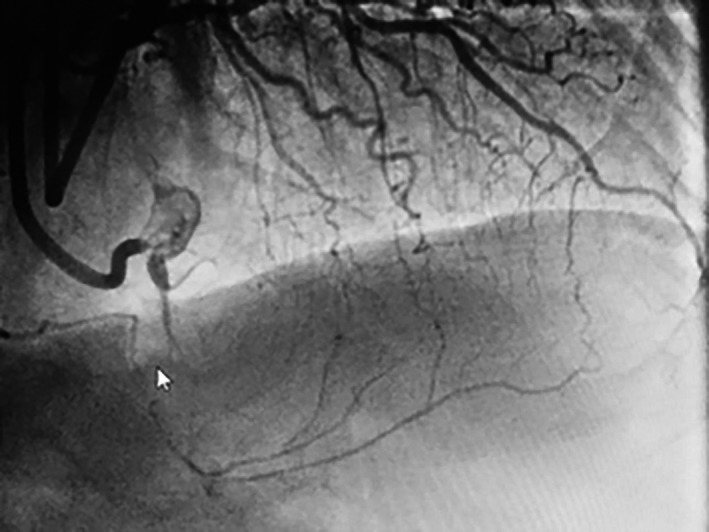
Total occlusion of the right coronary artery (RCA) with left to right collateral via septal arteries.

A 72‐year‐old male with hypertension, hyperlipidemia, and normal left ventricular ejection fraction presented with crescendo angina pectoris. Coronary angiography revealed two vessel diseases with a hemodynamically significant lesion in the first obtuse marginal (OM) branch and a chronic total occlusion (CTO) in the proximal right coronary artery (RCA). The RCA lesion had an ambiguous proximal cap, was greater than 30 mm in length, and had an extensive calcification. It filled through collaterals from the left coronary artery, via the septal perforators (Fig. 1). After the successful PCI to the OM branch, the patient was evaluated 2 weeks later to reassess the symptoms. Despite adequate medical therapy, he continued to have angina, which was of class 2 in severity as per Canadian Cardiovascular Society (CCS) and therefore accepted for angioplasty to the RCA.

**Figure 2:**
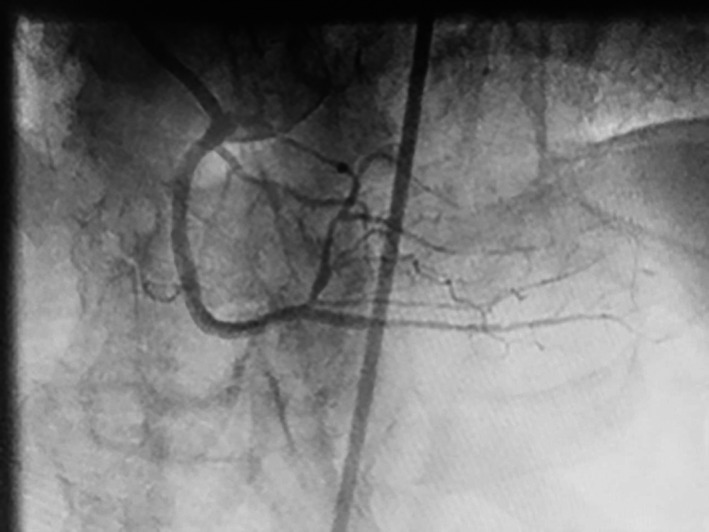
Successful stenting with three drug‐eluting stents.

For the procedure, we used bilateral femoral artery access with 8F sheaths. We intubated the RCA and the LM with 8F Amplatz left—AL 0.75 (Cordis, Diagnostic Solutions, Milpitas, CA) and 8F Extra Back Up—EBU 3.75 (Medtronic, Minneapolis, MN), respectively, for dual injection. Due to proximal cap ambiguity, only a brief anterograde attempt was made; given the lack of progress, we switched to retrograde approach. Via the distal septal, a 0.014″ Fielder FC guidewire (Asahi Intecc, Nagoya, Japan) with the aid of a Corsair 150‐cm microcatheter (Asahi Intecc, Nagoya, Japan) was used to access the posterior interventricular branch/posterior descending artery (PIV/PDA). Subintimal dissection with a knuckle wire in a retrograde approach was difficult due to the extremely fibrotic nature of the occlusion. Multiple 0.014″ Pilot 200 (Abbott Vascular, Santa Clara, CA) and Fielder XT (Asahi Intecc, Nagoya, Japan) guidewires were used to cross the occlusion, eventually obtaining an adequate position to perform a reverse CART procedure. After we predilated the subintimal space with a 3 × 15 mm Trek balloon (Abbott Vascular, Santa Clara, CA), we were able to access into the antegrade guide and then externalize a R350 wire (Vascular Solutions, Minneapolis, MN). Three drug‐eluting stents were used to stent from the ostial RCA to the RCA crux: 2.5 × 38 mm, 3.0 × 38 mm, 3.0 × 20 mm Promus Premier (Boston Scientific, Marlborough). Postdilatation was performed with 2.75 × 15 mm and 3.0 × 15 mm noncompliant Trek balloons (Abbott Vascular, Santa Clara, CA) (Fig. 2). There were no angiographic or clinical complications during the procedure. We maintained all major branches of the RCA. At the end of the procedure, a bedside echocardiogram was performed, which showed no evidence of mechanical complication or pericardial effusion.

**Figure 3:**
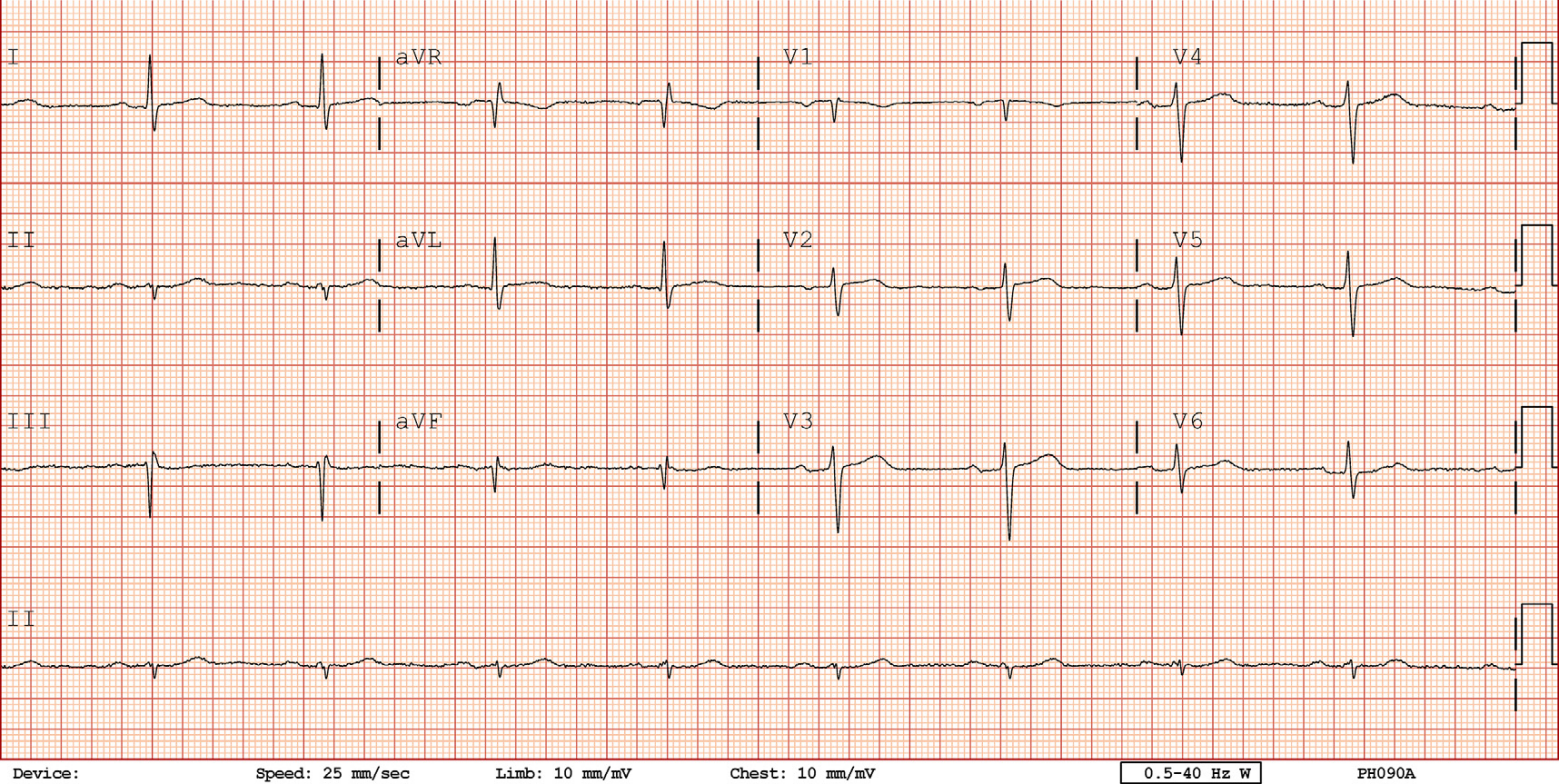
Two hours after procedure, under pleuritic pain no evidence of pericarditis changes.

Two hours after the PCI, the patient complained of chest pain, which was exacerbated by deep breathing and a supine position. The patient was stable without any changes in his vital signs or in his ECG (Fig. 3). The next morning the symptoms persisted, with a repeat echocardiogram confirming the absence of a pericardial effusion. A presumptive diagnosis of pericarditis was made, and the patient was discharged home with high dose of aspirin and colchicine, in addition to clopidogrel.

**Figure 4:**
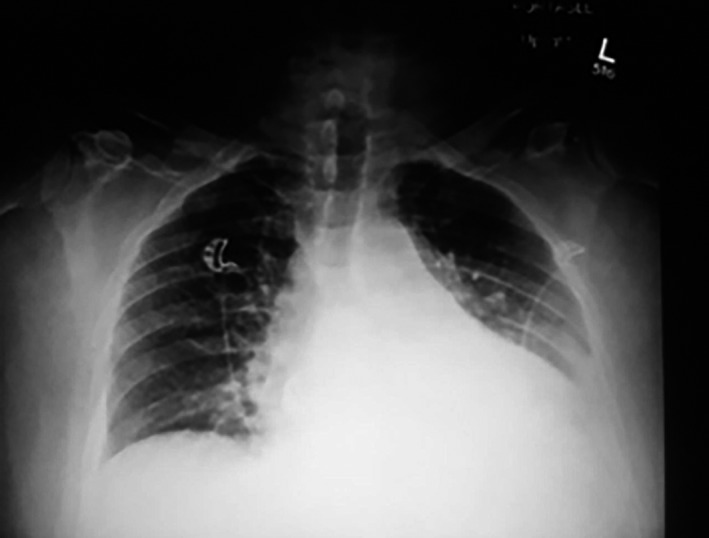
Chest X‐ray showing enlargement of the cardiac silhouette and left‐sided pleural effusion with accompanying atelectasis.

**Figure 5:**
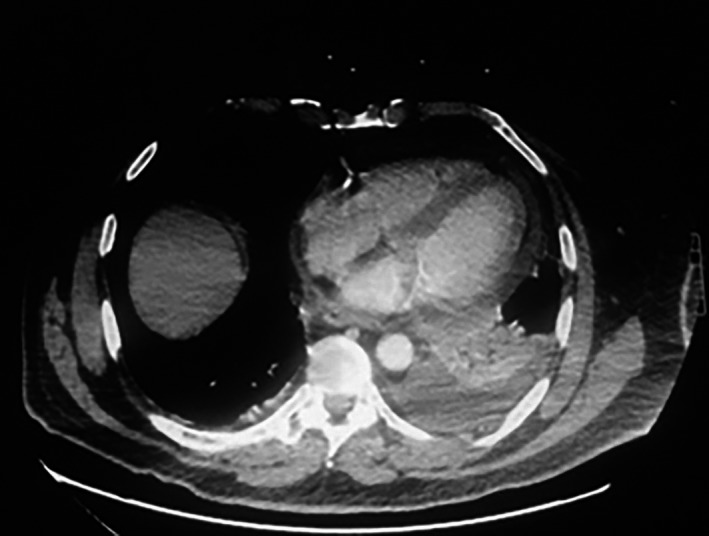
Pericardial effusion and bilateral pleural effusion.

Seven days postdischarge, he was re‐admitted with severe pleuritic chest pain and a low‐grade fever. The chest X‐ray demonstrated enlargement of the cardiac silhouette and left‐sided pleural effusion with accompanying atelectasis (Fig. 4). Chest computer tomography (CT) scan showed both a small pericardial effusion and bilateral pleural effusions (Fig 5). Over 1 L of serosanguinous pleural effusion was drained from the left chest. The effusion had exudative features. Echocardiography showed normal left ventricular systolic function, in addition to a small pericardial effusion posterior/lateral to the left ventricle with no cardiac chamber compression and left pleural effusion. ECG was unremarkable and unchanged from baseline. Blood tests were indicative of inflammation with elevations of white blood cell count (13.3 × 10^9^/L), serum C‐reactive protein (CRP) level (124 mg/L), and erythrocyte sedimentation rate (ESR) level (41 mm/HR), in addition to a hemoglobin drop to 102 g/L.

The patient was diagnosed with acute pleuropericarditis. He was treated with a single dose of 500 mg of hydrocortisone intravenously and then 60 mg/day of oral prednisone with a slow taper of 5 mg every 2 days. The day after steroid initiation, the patient's condition rapidly improved with resolution of the chest pain. One month after discharge, the patient was asymptomatic with no recurrent pleural effusion on chest X‐ray.

## Discussion

Post‐traumatic pericarditis is a subclassification of the PCIS. Post‐traumatic pericarditis can occur following blunt or penetrating trauma or maybe iatrogenic after intracardiac interventions such as percutaneous balloon pericardiotomy 6, pacemaker lead insertion, or radio‐frequency ablation 7. PCIS after PCI is most frequently described in cases complicated by either coronary perforation or dissection, although rare cases have occurred in uncomplicated cases 8, 9, 10.

The etiology of PCIS is believed to be secondary to an autoimmune phenomenon 1. The hypothesized pathophysiology is that the primary injury 11, 12, 13 releases pleuropericardial antigens into the circulation which stimulates an immune response involving the pleura, the pericardium, or both 14. The sequela is dependent on the level of circulating pleuropericardial antigen. High circulating levels of antigens can induce autoantibodies and form immune complexes that deposit in the pleura, lungs, pericardium, and joints, provoking inflammation similar to other immune complex diseases 11. The latent period between this initial event and the first clinical manifestation, together with the elevation of the inflammatory markers and the remarkable response to anti‐inflammatory drugs (NSAIDs, corticosteroids, colchicine), supports the autoimmune theory 1. The occurrence of early onset PCIS suggests the concomitant importance of direct trauma to either the pericardium or the pleura.

In the 2015 ESC guidelines, a diagnosis of PCIS requires prior injury of the pericardium and/or myocardium and at least two of the following (1) fever without alternative causes, (2) pericarditic or pleuritic chest pain, (3) pericardial or pleural rubs, (4) evidence of pericardial effusion, or (5) pleural effusion with elevated CRP 1. PCIS most commonly occurs after a latent period of approximately 7–20 days from the inciting event 1, 7, 12. However, there are rare reports of early PCIS, occurring as soon as 4 hours after injury 5, 9. The early presentation of PCIS is thought to be due to two potential processes. The first theory is that the autoimmune reaction is accelerated by a viral infection or by the presence of blood in the pericardial space secondary to the trauma 11. The second theory is based on preimmunization process. According to Setoyama et al., a prior stimulation of the immune system by a recent myocardial injury could explain the rapid onset of PCIS. After pre‐exposure, even minor injuries occurring at a later time might trigger the creation of immune complexes and the early activation of the inflammatory pathway.

Our patient's first symptom was pleuritic chest pain, which started almost immediately after the procedure. This was likely attributable to acute injury to the visceral pericardium with an auto‐immune response that occurred shortly after the initial event, explaining the subsequent second admission. Explanations for the initial pleuritic pain could have been due to the presence of blood in the pericardium from an aggressive subintimal dissection and microtrauma with the knuckle wire. Although serial postprocedural echocardiograms did not find any evidence of pericardial effusion, at his second admission, pericardial and pleural effusions were demonstrated on imaging. A communication between the pericardium and the pleura from an unrecognized microperforation cannot be discounted. Another possible explanation is a subadventitial hematoma from the aggressive dissection led to visceral pericardium inflammation or to a subclinical hematoma, without any blood entering the pericardial space, therefore not appearing on our echography studies.

Seven days later on his second admission, he was found to have pleural and pericardial effusions along with elevated inflammatory markers and low‐grade fever. This satisfied the ESC criteria for PCIS. The dramatic clinical improvement and the decrease in the inflammatory markers after the administration of corticosteroids strongly suggest an immune‐mediated etiology in this case.

## Conclusion

In summary, we present a rare case of acute pleuropericarditis occurring after a successful complex PCI.

## Ethics, Consent, and Permission

As per the institutional requirements for the Sunnybrook Health Sciences Center Research ethics board, the need for ethics review was waived.

## Consent to Publish

We have obtained consent from the patient for publication of this report.

## Conflict of Interest

None declared.

## Authorship

GEG: interpreted results and drafted the manuscript. HCW: conceived idea and revised the manuscript critically for content.
